# RETREATMENT WITH SURFACTANT IN VERY LOW BIRTH WEIGHT PRETERM INFANTS: RISK PREDICTORS AND THEIR INFLUENCE ON NEONATAL OUTCOMES

**DOI:** 10.1590/1984-0462/2021/39/2019360

**Published:** 2020-11-16

**Authors:** Walusa Assad Gonçalves Ferri, Adriana Carnevale da Silva, Eliana Motta Fernandes Sacramento, Cristina Calixto, Davi Casale Aragon, Jamil Pedro de Siqueira Caldas

**Affiliations:** a Universidade de São Paulo, Ribeirão Preto, SP, Brazil.; b Universidade Estadual de Campinas, Campinas, SP, Brazil.

**Keywords:** Pulmonary surfactant, Retreatment, Risk factors, Preterm infant, Bronchopulmonary dysplasia, Surfactante pulmonar, Retratamento, Fatores de risco, Recém-nascido prematuro, Displasia broncopulmonar

## Abstract

**Objective:**

To assess clinical predictors and outcomes associated to the need for surfactant retreatment in preterm infants.

**Methods:**

Retrospective cohort study, including very low birth weight preterm infants from January 2006 to December 2015 who underwent surfactant replacement therapy. Beractant was used (100 mg/kg), repeated every six hours if FiO_2_ ≥0.40. The subjects were classified into two groups: single surfactant dose; and more than one dose (retreatment). We evaluated maternal and neonatal predictors for the need of retreatment and neonatal outcomes associated to retreatment.

**Results:**

A total of 605 patients (44.5%) received surfactant; 410 (67.8%) one dose, and 195 (32.2%) more than one dose: 163 (83.5%) two doses and 32 (16.4%) three doses. We could not find clinical predictors for surfactant retreatment. Retreatment was associated to a greater chance of BPD in infants >1000 g (RR 1.78; 95%CI 1.30‒2.45) and ≤1000 g (RR 1.33; 95%CI 1.04‒1.70), in infants with gestational age<28 weeks (RR 1.56; 95%CI 1.12‒2.18) and ≥28 weeks (RR 1.50; 95%CI 1.17‒1.92), in neonates with early sepsis (RR 1.48; 95%CI 1.20‒1.81), and in infants not exposed to antenatal corticosteroids (RR 1.62; 95%CI 1.20‒2.17)

**Conclusions:**

We could not find predictor factors associated to surfactant retreatment. The need for two or more doses of surfactant was significantly related to bronchopulmonary dysplasia.

## RESUMO

**Objetivo:**

Avaliar preditores clínicos e resultados associados à necessidade de retratamento com surfactante.

**Métodos:**

Coorte retrospectiva com prematuros de muito baixo peso, no período de janeiro de 2006 a dezembro de 2015, em uso de terapia de reposição de surfactante. O surfactante utilizado foi beractante (100 mg/kg), repetido a cada seis horas se FiO_2_≥0.40. Foram analisados dois grupos: dose única de surfactante e mais de uma dose (retratamento). Foram avaliados preditores maternos e neonatais para retratamento e resultados neonatais.

**Resultados:**

605 pacientes (44,5%) receberam surfactante; 410 (67,8%) uma dose e 195 (32,2%) mais de uma dose: 163 (83,5%) duas doses e 32 (16.4%) três doses. Não foram encontrados fatores associados ao retratamento com surfactante. A displasia broncopulmonar (DBP) foi associada ao retratamento (p<0.01). A presença de retratamento aumentou a chance de ocorrência de DBP em neonatos >1000 g (RR 1,78; IC95% 1,30‒2,45) e ≤1000 g (RR 1,33; IC95% 1,04‒1,70), em recém-nascidos com idade gestacional <28 semanas (RR 1,56; IC95% 1,12‒218) e ≥28 semanas (RR 1,50; IC95% 1,17‒1,92), naqueles com sepse precoce (RR 1,48; IC95% 1,20‒1,81), e nos que não foram expostos ao corticoide antenatal (RR 1,62; IC95% 1,20‒2,17).

**Conclusões:**

Não encontramos fatores preditores associados à necessidade de retratamento. A necessidade de duas ou mais doses de surfactante está associada à displasia broncopulmonar.

## INTRODUCTION

The advances in mechanical ventilation, nutrition, and behavioral adaptation strategies have produced a significant increase in survival rates of preterm infants and is, currently, approximately 11.3% in the USA, which increased the rates of morbidity in preterm infants, mainly due to respiratory aspects.^1^ ^,^ ^2^

Treatment with exogenous surfactant is currently the major pillar of RDS management. It has proved to be efficient in both clinical and experimental conditions, and it may increase blood oxygen levels and pulmonary compliance within minutes after its administration.^3^ ^,^ ^4^ ^,^ ^5^ The use of surfactant reduces neonatal mortality. However, even today, there are still some controversies regarding the administration and retreatment.^6^ ^,^ ^7^ ^,^ ^8^ ^,^ ^9^

After surfactant administration, there is often an initial clinical improvement, which can be followed, hours later, by deterioration of lung function, requiring retreatment.^10^ ^,^ ^11^ Retreatment is recommended according to the severity of the respiratory failure, and it is currently endorsed when there is a need for a fraction of inspired oxygen (FiO_2_) of over 0.40 for patients older than 26 weeks, and over 0.30 for patients younger than 26 weeks to obtain proper oxygenation.^12^

Retreatment is usually necessary for patients who show higher surfactant consumption related to a more severe pulmonary condition, like those with lower birth weight, a more severe radiological condition, and those with a shorter desaturated-phosphatidylcholine (DSPC) half-life.^13^ However, the literature poorly describes associations with the need for retreatment and outcomes.^13^ ^,^ ^14^ Some studies described a decrease in bronchopulmonary dysplasia and mortality rates in occasions where there is also a decrease in retreatment, but they did not describe measures of association between such occurrences.^6^ ^,^ ^7^ ^,^ ^8^ Knowledge of variables associated with the need for surfactant retreatment may help clinician in the neonatal respiratory management.

Thus, the objective of the present study was to analyze the predictors associated to the need for surfactant retreatment, as well as its effects on clinical outcomes in very low birth weight (VLBW) preterm infants during hospitalization.

## METHOD

This cohort study is a retrospective analysis of prospectively collected data, using the hospital database. All very low birth weight (VLBW) preterm infants registered in the database between January 2006 and December 2015 were included in this study. We have excluded all patients with major congenital malformations and death in the delivery room. In order to assess bronchopulmonary dysplasia, we have also excluded those who died before 36 weeks of postmenstrual age. Patients were divided into two groups for comparison. The first group consisted of those who had received only one surfactant dose. The second was the retreatment group, with neonates who received two or more surfactant doses. Maternal and neonatal variables were evaluated to determine the clinical predictors of surfactant retreatment, and to analyze the effect of retreatment on neonatal outcomes (bronchopulmonary dysplasia, pulmonary hemorrhage, severe peri-intraventricular hemorrhage, pneumothorax and hospital death).

The first dose of surfactant was administered when patients required FiO₂ equal to or over 0.40 to obtain target oxygen saturation between 90‒94%. One or two additional doses were administered every six hours if FiO_2_ remained equal to or over 0.40 to maintain oxygen saturation in the targets established by the unit (92‒94%). All infants received bovine (Beractant - Survanta^®^ - AbbVie Inc. USA) - 100 mg/kg for both the first administration and retreatment doses. There was no prophylactic treatment with surfactant. Surfactant administration was performed in bolus, via orotracheal cannula.

The mechanical ventilation protocol was similar throughout the period, and pressure limited and time cycled ventilatory mode was used. Respirators used in the early years of the series were predominantly unsynchronized equipment and, in the following years, synchronized respirators with a target volume of 6 mL/kg were used. The clinical protocols in the delivery room followed the guidelines of the Neonatal Resuscitation Program of the Brazilian Society of Pediatrics. CPAP in delivery room has been used since 2010.

The maternal variables analyzed were arterial hypertension and antenatal use of steroids, defined as the antenatal administration of at least one betamethasone and/or dexamethasone dose to the mother during pregnancy at any time before delivery. Neonatal variables included birth weight, gestational age (the best estimate between amenorrhea and the one determined with early echography or the new Ballard score), gender, need for resuscitation in the delivery room (defined by need for positive airway pressure by mask and/or endotracheal tube), temperature at admission to the neonatal care unit, SNAPPE II score (<20 and ≥20), time after birth at surfactant administration and early or late clinical sepsis (blood-culture-proven episodes or presumed neonatal sepsis, defined by hematological criteria, associated to worsening in the clinic parameters and the use of antibiotics for more than 72 hours.

The assessed neonatal outcomes were pulmonary hemorrhage, peri-intraventricular hemorrhage diagnosed by cranial ultrasound, classified according to Papille et al.,^15^ Bell stages II or III necrotizing enterocolitis,^16^ pneumothorax, bronchopulmonary dysplasia (defined by the need for oxygen use at 36 weeks corrected gestational age),^17^ and death. Mechanical ventilation and hospitalization times have also been evaluated.

We used Chi-square test for the comparisons of categorical variables levels, and the Student’s t-test or ANOVA for the comparisons of means of continuous variables. To quantify the associations between surfactant usage and possible predictors, we fitted simple log-binomial regression models. In order to associate neonatal outcomes to the need for surfactant retreatment (separately composed with birth weight, gestational age, early sepsis and antenatal steroids) we fitted simple and multiple log-binomial regression models, considering maternal hypertension, early sepsis, and type of delivery as covariates for birth weight/surfactant retreatment; SNAPPEII, maternal hypertension, early sepsis and type of delivery for GA/surfactant retreatment, and antenatal steroids/ surfactant retreatment; SNAPPEII, maternal hypertension and type of delivery for sepsis/surfactant retreatment. For the evaluation of mechanical ventilation and hospitalization time variables, we generated Kaplan-Meier graphs and compared the curves with the Wilcoxon test. In the analysis of predictors and outcomes, the level of significance was set at 5% and the software used was SAS 9.4. The study was approved by the Ethics Committee for research, protocol No. 1.018.827 of 04/09/2015.

## RESULTS

There were 1.481 VLBW preterm infants admitted (93.2% inborn) to the institution between January 2006 and December 2015. We excluded 90 patients who died in the delivery room, 32 patients who presented significant malformations and 210 patients who died before 36 weeks of life (only to assess bronchopulmonary dysplasia). Thus, there were 1,359 eligible patients for retreatment evaluation. Of the total, 754 (55.5%) did not require surfactant, and 605 (44.5%) received at least one surfactant dose; namely 410 (67.8%) received only one dose, and 195 (32.2%) required additional doses - 163 (83.5%) received two doses, and 32 (16.4%) received three doses. The mean age for the first surfactant dose administration was 5 hours and 30 minutes of life. No difference was noted between newborns that received single or multiple doses (retreatment) both for birth weight (911±300 × 953±300 g; p=0.11) and gestational age (27.5±2.6 × 28.0±2.6 weeks; p=0.07).

For better characterization of surfactant retreatment predictors, we evaluated birth weight, gestational age, maternal hypertension, antenatal steroids, gender, and resuscitation in the delivery room, and NICU temperature at admission. These variables were not associated to the need for retreatment. (Table 1)

**Table 1:** Clinical predictors for the need for surfactant retreatment in very low birth weight preterm infants.

	Surfactant		RR (95%CI)
	2 or more	1 dose	
Birth weight			
≤1000 g	127 (34.7)	239 (65.3)	1.21 (0.9-1.63)
>1000 g	68 (28.5)	171 (71.6)	reference
Gestational age			
<28 weeks	96 (35.6)	174 (64.4)	1.2 (0.95-1.51)
≥28 weeks	99 (29.6)	235 (70.4)	reference
Maternal hypertension			
No	123 (30.2)	284 (69.8)	reference
Yes	69 (36.9)	118 (63.1)	1.22 (0.96-1.55)
Antenatal steroids			
No	94 (31.9)	201 (68.1)	reference
Yes	97 (32.2)	204 (67.8)	1.01 (0.8-1.28)
Gender			
Male	107 (33.7)	211 (66.4)	1.10 (0.86-1.38)
Female	88 (30.7)	199 (69.3)	reference
Resuscitation			
No	7 (25.0)	21 (75.0)	reference
Yes	184 (32.6)	381 (67.4)	1.30 (0.68-2.50)
Temperature at NICU admission			
≤35.9	73 (31.3)	160 (68.7)	1.28 (0.84-1.97)
36 to 36.4	25 (30.9)	56 (69.1)	1.26 (0.77-2.09)
36.5 to 37.5	20 (24.4)	62 (75.6)	reference
>37.5	3 (50.0)	3 (50.0)	2.05 (0.85-4.97)
SNAPPE II			
<20	52 (31.5)	113 (68.5)	reference
≥20	142 (33.1)	287 (66.9)	1.05 (0.80-1.37)
Time of life at 1^st^ dose			
≤3h	105 (35.4)	192 (64.7)	1.21 (0.96-1.53)
>3h	90 (29.2)	218 (70.8)	reference
Early sepsis			
No	17 (29.3)	41 (70.7)	reference
Yes	168 (34.2)	323 (65.8)	1.17 (0.77-1.77)

n (%); RR: relative risk; 95%CI: 95% confidence interval; NICU: Neonatal Intensive Care Unit; SNAPPE-II: Score for Neonatal Acute Physiology with Perinatal Extension-II.

Regarding clinical outcomes during hospitalization, we found that the need for retreatment was significantly associated to the occurrence of bronchopulmonary dysplasia (p<0.01). Other variables, such as pulmonary hemorrhage (p=0.53), intraventricular hemorrhage grade 3‒4 (p=0.71), necrotizing enterocolitis (p=0.24), pneumothorax (p=0.76) and death (p=0.10) were not related to surfactant retreatment (Table 2).

**Table 2:** Clinical outcomes according to the number of surfactant doses in very low birth weight preterm infants.

Clinical outcomes	Surfactant			
	1 dose (n=410)	2 doses (n=163)	3 doses (n=32)	p-value
Pulmonary hemorrhage	85 (20.3%)	37 (22.6%)	6 (18.7%)	0.53
IVH 3 - 4	62 (15.1%)	28 (17.1%)	6 (18.7%)	0.71
Necrotizing Enterocolitis	37 (9.0%)	11 (6.7%)	5 (15.6%)	0.24
Pneumothorax	73 (17.8%)	33 (20.2%)	5 (15.6%)	0.76
BPD	116 (28.3%)	57 (34.9%)	18 (56.2%)	<0.01
Death	142 (34.6%)	70 (42.9%)	11 (34.3%)	0.10

BPD: bronchopulmonary dysplasia; IVH: Intraventricular hemorrhage grades 3 or 4.

We found a positive association between the need for surfactant retreatment and the development of bronchopulmonary dysplasia. Table 3 presents the results of log-binominal regression aiming to verify the relation between bronchopulmonary dysplasia and retreatment for patients with variables that could be related to the development of the disease.

**Table 3:** Log-binominal regression model associating retreatment, maternal and neonatal clinical characteristics, and need for oxygen at 36 weeks corrected age.

	Outcome: need for oxygen at 36 weeks corrected age		RR adj (95%CI)
	Yes	No	
Birth weight ≤1000 g/surfactant			
1 dose (n=120)	63 (52.5)	57 (47.5)	reference
2 doses (n=58)	39 (67.2)	19 (32.8)	1.33 (1.04-1.70)
Birth weight >1000 g/surfactant			
1 dose (n=152)	50 (32.9)	102 (67.1)	reference
2 doses (n=57)	36 (63.2)	21 (36.8)	1.78 (1.30-2.45)
Gestational age <28 weeks/surfactant			
1 dose (n=72)	33 (45.8)	39 (54.2)	reference
2 doses (n=32)	23 (71.9)	9 (28.1)	1.56 (1.12-2.18)
Gestational age ≥28 weeks/surfactant			
1 dose (n=199)	80 (40.2)	119 (59.8)	reference
2 doses (n=83)	52 (62.7)	31 (37.4)	1.50 (1.17-1.92)
Sepsis-YES/surfactant			
1 dose (n=221)	100 (45.3)	121 (54.8)	reference
2 doses (n=107)	70 (65.4)	37 (34.6)	1.48 (1.20-1.81)
Antenatal steroids-NO/surfactant			
1 dose (n=117)	49 (41.9)	68 (58.1)	reference
2 doses (n=52)	35 (67.3)	17 (32.7)	1.62 (1.20-2.17)
Antenatal steroids-YES/surfactant			
1 dose (n=152)	63 (41.5)	89 (58.6)	reference
2 doses (n=60)	38 (63.3)	22 (36.7)	1.42 (1.08-1.86)

RR adj: adjusted relative risk considering maternal hypertension, early sepsis and type of delivery as covariates for birth weight; SNAPPEII, maternal hypertension, early sepsis, and type of delivery for GA and antenatal steroids; SNAPPEII, maternal hypertension and type of delivery for sepsis; 95%CI: 95% confidence interval.

When the risk of BDP was analyzed in relation to single or multiple surfactant doses adjusted to some maternal or neonatal variables, retreatment was associated to a greater chance of BPD in infants >1000 g (RR 1.78; 95%CI 1.30-2.45) and ≤1000 g (RR 1.33; 95%CI 1.04-1.70), in infants with gestational age<28 weeks (RR 1.56; 95%CI 1.12-2.18) and ≥28 weeks (RR 1.50; 95%CI 1.17-1.92), in neonates with early sepsis (RR 1.48; 95%CI 1.20-1.81), and in infants not exposed to antenatal corticosteroids (RR 1.62; 95%CI 1.20‒2.17)

We did not observe a statistically significant association between the need of surfactant retreatment and mechanical ventilation duration (p=0.06) or with hospital length of stay (p=0.73).

## DISCUSSION

Surfactant replacement therapy is an essential strategy for respiratory distress syndrome treatment for preterm infants. Nevertheless, some doubts persist regarding this treatment, for example, who are the candidates to retreatment and what is the impact of the need for retreatment on the patient’s evolution.

We did not identify predictors for surfactant retreatment. Cogo et al. demonstrated that the lower the birth weight, the higher the chance of the need for retreatment. They also found others predictors for surfactant retreatment, such as worse radiological severity score (grade 3 and 4 *versus* 1 and 2 - OR 4.0; 95%CI 1.2-14.3), initial surfactant dose (100 *versus* 200 mg/kg - OR 7.3; 95%CI 1.8-30.1), and ventilation strategy (conventional mechanical ventilation *versus* high-frequency oscillatory ventilation - OR 9.4; 95%CI 1.6-50.0).^13^ In our study, due to its retrospective design, it was not possible to retrieve data about radiological severity score and ventilation strategies.

We did not find an association between the need for retreatment and other outcomes, such as grades 3 and 4 peri-intraventricular hemorrhage, necrotizing enterocolitis, and death. There was also no association with length of hospital stay (p=0.06) and mechanical ventilation days (w=0.76). Kattwinkel et al.^18^ and Cogo et al.^19^ also did not observe retreatment association with these outcomes.^18^ ^,^ ^19^

Our study showed that the need for retreatment was associated to BPD, and there is no higher risk of pneumothorax, pulmonary hemorrhage, severe IVH, necrotizing enterocolitis, and death in the retreatment group. A review by Cochrane in 2009 showed that the administration of multiple surfactant doses resulted in a lower risk of pneumothorax and death. However, contrasting with our study, in most evaluated studies in the review by Cochrane, the additional doses of surfactant were indicated with FiO2 under 0.3, and the included patients were more mature and less exposed to antenatal steroids.^14^ Dunn et al. evaluated the effect of a single dose compared to multiple surfactant doses in neonates with 30‒36 weeks of gestational age and mild and moderate respiratory distress. They found improved gas exchange but no significant difference in bronchopulmonary dysplasia rates and death, using bovine surfactant. However, the criterion used for retreatment was an increase in FiO_2_ of only 0.1 after the initial dose.^20^ Nevertheless, the studies presented above are older, with less use of antenatal steroids and more mature patients.^14^ ^,^ ^20^

Similar studies show higher retreatment rates, approximately from 48.9 to 62.5%, using 100 mg/kg as the initial dose. However, surfactant recommendations were different: Speer et al. and Ramanathan et al. used FiO_2_ ≥0.3 as a retreatment recommendation indication. In our study, we used FiO_2_ ≥0.4 for indication, which may explain a slightly lower retreatment rate.^7^ ^,^ ^8^

In our sample, the need for retreatment was considered an undesirable event, as we have found an association with bronchopulmonary dysplasia, even after adjusting for gestational age and SNAPPE II. When we evaluated the association between the number of surfactant doses, the development of bronchopulmonary dysplasia, and the clinical characteristics related to poor pulmonary prognosis, septic patients and those who were not exposed to antenatal steroid and needed retreatment showed a significant increase in the risk of developing bronchopulmonary dysplasia, with an adjusted relative risk of 1.48 (95%CI 1.20‒1.81) and 1.62 (95%CI 1.20‒2.17), respectively. Interestingly, in the present study, preterm infants with birth weight>1000 g presented a higher relative risk of BPD. This finding is possibly attributed to the presence of more severe lung disease in larger neonates and, consequently, more chances of developing dysplasia. Nevertheless, due to the retrospective nature of our study, it was not possible to identify the severity of lung disease.

There are some possibilities of interpretation concerning the association found in this study between the need for retreatment and bronchopulmonary dysplasia. The repeated doses of surfactant probably were not the factor responsible for BPD, because it has already been demonstrated in the literature that there is no association between multiple doses of surfactant and adverse outcomes.^14^ An insufficient initial replacement dose and the type of surfactant used were a possible explanation to an association between surfactant retreatment and BPD. A recent Cochrane meta-analysis evaluated three treatment studies comparing bovine to porcine surfactant preparations and it showed a significant increase in the risk of death or oxygen requirement at 36 weeks postmenstrual age (typical RR 1.30; 95%CI 1.04‒1.64) when the bovine product was used (448 infants; moderate quality evidence).^6^ Late administration in our study (more than 5 hours) is another important discussion point. Although there is a strong recommendation to use surfactant before two hours of life, the pragmatic design of our study could not differentiate if the delay in treatment occurred because the infant did not fulfill criteria for administration of the drug or if there was another cause for this event. Other authors demonstrated a decrease in BPD frequency with the administration of higher doses, different surfactant types (porcine surfactant), and early administration to surfactant replacement.^7^ ^,^ ^8^ ^,^ ^13^ ^,^ ^19^ ^,^ ^21^ ^,^ ^22^ ^,^ ^23^ Moreover, in other studies in the literature, using wider FiO_2_ for indicating surfactant retreatment, such as the use of FiO_2_<0.3 and early rescue, multiple doses of surfactant were not associated to adverse outcomes.^7^ ^,^ ^8^ ^,^ ^13^ ^,^ ^19^ ^,^ ^21^ ^,^ ^22^ ^,^ ^23^

Therefore, we considered that the value of FiO_2_>0.4 for indicating the retreatment of patients under 26 weeks might have favored the association with bronchopulmonary dysplasia. Lim et al. demonstrated that the use of surfactant with FiO_2_>0.3 in 25‒28 weeks gestational age neonates is related to worse outcomes (bronchopulmonary dysplasia, death, necrotizing enterocolitis, and other morbidities) and the more recent European consensus guideline on the management of respiratory distress syndrome suggests surfactant administration when FiO_2_>0.3 is needed in all gestational ages.^23^ ^,^ ^24^

An important limitation of this study is the fact that it was not possible to report details about the mechanical ventilation in each single patient; however, in the hospital evaluated, every patient was ventilated in the pressure-limited mode and time cycled during the study period. Mechanical ventilation duration was similar (p=0.06) between preterm infants who received single or multiple surfactant treatments. CPAP in delivery rooms was introduced in 2010, and that was why we could not evaluate this important ventilatory intervention and its implication in respiratory outcomes in VLBW infants. Besides that, the pulmonary disease severity was not described. Considering that the present study is a pragmatic cohort, variables not collected at the time cannot be adequately evaluated. However, despite these limitations, the study has important translational aspects for neonatal units, since surfactant replacement therapy is an essential therapeutic strategy in neonatal units.^25^ ^,^ ^26^

In conclusion, we did not identify predictor factors to surfactant retreatment. Retreatment has been found to be associated with bronchopulmonary dysplasia.
